# The Influence of Prolonged Acetylsalicylic Acid Supplementation-Induced Gastritis on the Neurochemistry of the Sympathetic Neurons Supplying Prepyloric Region of the Porcine Stomach

**DOI:** 10.1371/journal.pone.0143661

**Published:** 2015-11-25

**Authors:** Katarzyna Palus, Jarosław Całka

**Affiliations:** Department of Clinical Physiology, Faculty of Veterinary Medicine, University of Warmia and Mazury in Olsztyn, Olsztyn, Poland; Max-Delbrück Center for Molecular Medicine (MDC), GERMANY

## Abstract

This experiment was designed to establish the localization and neurochemical phenotyping of sympathetic neurons supplying prepyloric area of the porcine stomach in a physiological state and during acetylsalicylic acid (ASA) induced gastritis. In order to localize the sympathetic perikarya the stomachs of both control and acetylsalicylic acid treated (ASA group) animals were injected with neuronal retrograde tracer Fast Blue (FB). Seven days post FB injection, animals were divided into a control and ASA supplementation group. The ASA group was given 100 mg/kg of b.w. ASA orally for 21 days. On the 28^th^ day all pigs were euthanized with gradual overdose of anesthetic. Then fourteen-micrometer-thick cryostat sections were processed for routine double-labeling immunofluorescence, using primary antisera directed towards tyrosine hydroxylase (TH), dopamine β-hydroxylase (DβH), neuropeptide Y (NPY), galanin (GAL), neuronal nitric oxide synthase (nNOS), leu 5-enkephalin (LENK), cocaine- and amphetamine- regulated transcript peptide (CART), calcitonin gene-related peptide (CGRP), substance P (SP) and vasoactive intestinal peptide (VIP). The data obtained in this study indicate that postganglionic sympathetic nerve fibers supplying prepyloric area of the porcine stomach originate from the coeliac-cranial mesenteric ganglion complex (CCMG). In control animals, the FB-labelled neurons expressed TH (94.85 ± 1.01%), DβH (97.10 ± 0.97%), NPY (46.88 ± 2.53%) and GAL (8.40 ± 0.53%). In ASA group, TH- and DβH- positive nerve cells were reduced (85.78 ± 2.65% and 88.82 ± 1.63% respectively). Moreover, ASA- induced gastritis resulted in increased expression of NPY (76.59 ± 3.02%) and GAL (26.45 ± 2.75%) as well as the novo-synthesis of nNOS (6.13 ± 1.11%) and LENK (4.77 ± 0.42%) in traced CCMG neurons. Additionally, a network of CART-, CGRP-, SP-, VIP-, LENK-, nNOS- immunoreactive (IR) nerve fibers encircling the FB-positive perikarya were observed in both intact and ASA-treated animals. The results of this study indicate involvement of these neuropeptides in the development or presumably counteraction of gastric inflammation.

## Introduction

The past thirty years have shown increasingly rapid advances in studies of innervation of the gastrointestinal tract. In general, the stomach and gut are innervated both by neurons found within intramural ganglia and thus belong to enteric nervous system (ENS) [1, 2], as well as by extrinsic cell bodies originating in sympathetic, parasympathetic and sensory ganglia [3–5]. Recent investigations have revealed that sympathetic ganglia are not only centers of nervous integration but also the possession of important properties by their neurons. Among others they include convergence of central impulses, projection of visceral impulses at the pre- and post-synaptic levels, accessing/allowing for the central fibers of visceral protection and pacemaker activity [6, 7]. However, sympathetic postganglionic neurons that supply the gastrointestinal tract do not directly influence on its functions but exert their effects through the ENS [8, 9], or constrict the arteries that supply the digestive organ [10]. Furthermore, the stomach function is mediated and modulated by plenty of neuronal transmitters and neuropeptides, which play a role in the regulation of motility, acid secretion, hormone release, local blood flow and mucosal defence mechanisms [3].

There is a large volume of published studies describing sympathetic innervation of the stomach, based mainly on small laboratory animals, such as rat [10–12], mouse [13, 14], guinea pig [15, 16], rabbit [17] or domestic animals, such as dog [7] and cat [18, 19]. The authors report that prevertebral ganglia eg. celiac ganglion constitute the main source of postganglionic sympathetic innervation of the abdominal viscera. Whereas only single perikarya were found in paravertebral ganglia eg. sympathetic chain ganglia [16, 20]. Hitherto, relatively little is known about innervation of stomach in the domestic pig, which closely resemble that in human in respect of anatomical and physiological characteristic [21, 22]. Previous studies in the field describe only the extrinsic innervation of small and large intestine [4, 20] or focus on the enteric nervous system [23, 24].

The autonomic nervous system is characterized by high plasticity in response to various pathological stimuli, and the ability to adapt to the changing environmental conditions [25, 26]. This adaptation involves changing in the chemical phenotype of neurons by increased expression of some neurotransmitters and reduced of others or activation of expression of previously inactive genes [26, 27]. In recent years, there has been an increasing amount of literature describing changing of chemical coding of sympathetic neurons supplying the gastrointestinal tract during ileitis [20], proliferative enteropathy [28], colitis [4] and axotomy [29–31]. Additionally, some authors suggest that sympathetic neurons not only change their chemical characteristic, but also exhibit the ability to regenerate [32]. Interestingly, some authors suggest that sympathetic nervous system play a role as a modulator of gastrointestinal inflammation, because sympathetic neurons supply lymphoid tissues. Moreover, the presence of the receptors for sympathetic neurotransmitters in immune cells have been confirmed [33].

Acetylsalicylic acid, well known as aspirin (ASA), is one of the most commonly used non-steroidal anti-inflammatory drugs (NSAIDs) worldwide and is especially valued for its therapeutic properties. The benefits of the wide therapeutic options, such as prevention of cardiovascular diseases, treatment of symptoms of variety of inflammatory conditions, antitumor activity, or pain relief have been known for many years [34]. Aspirin prevents prostaglandin synthesis by inhibiting the enzyme cyclooxygenase (COX) via irreversible acetylation of the hydroxyl group of 1 serine residue [35, 36]. COX called also as prostaglandin endoperoxidase synthase is a membrane—bound hemoprotein and glycoprotein enzyme that catalyzes conversion of cell membrane phospholipids, involved in the synthesis of prostanoids comprising: prostaglandin (PG), prostacyclin (PGI) and the thromboxanes (TXA) and exists as 3 isoforms (COX-1, -2 and -3) [34, 37]. Eicosanoids, derived from a reaction catalyzed by COX-1, are involved in platelet aggregation, protection of the gastric mucosa, and many other physiological processes, whereas those formed with the participation of COX-2 are involved only in development of the inflammatory response. COX-3 is a post-transcriptional modification of COX-1, occurring in the central nervous system [38].

Aspirin is a non-selective NSAID and inhibits the enzymatic activity of COX-1 several hundred times more effectively, compared with the COX-2 [38, 39]. Indeed, its action results in both beneficial (antipyretic and anti-inflammatory) and toxic (gastrointestinal injury) effects [40]. Even small dose of aspirin induces superficial injury in gastric epithelium and leads to abnormal ion flux with increased H^+^ back diffusion. Furthermore, the inhibition of COX-1, leading to deficiency of prostaglandins (PGs) in gastric and intestinal mucosa, is accepted as the main mechanism of mucosal damage that include bleeding, erosions and ulceration [41]. These harms occur most frequently in human antrum and prepyloric area, although they can also be seen in the proximal part of duodenum [34]. Moreover, decreased level of prostaglandin E2 (PGE2) exposes the mucous membrane to damage caused by hydrochloric acid and bile, and reduces the ability of regeneration of mucosal cells by decreasing release of mucus, an inhibition of surfactants and phospholipids synthesis, reduction in secretion of HCO_3_ and disorders of blood flow in the microcirculation [40, 42]. Currently, the influence of aspirin—induced gastritis on adaptive processes and neurochemical properties of sympathetic neurons supplying stomach is rather fragmentary.

Therefore, this experiment was designed to establish: 1) the localization and distribution of sympathetic neurons supplying prepyloric area of the stomach in domestic pig; 2) the neurochemical phenotype of traced perykarya in physiological state; 3) possible changes in neurochemical coding of traced neurons during gastritis induced by prolonged acetylsalicylic acid supplementation.

## Materials and Methods

### Ethics Statement

The experimental procedure including animal euthanasia was approved by the Local Ethical Commission for Experiments on Animals at the University of Warmia nad Mazury in Olsztyn (Permit Numbers 05/2010). All surgery was performed under sodium thiopental anaesthesia, and all possible efforts were made to minimize animal suffering.

### Animals and surgical procedures

The study was performed on ten juvenile female pigs of the Large White Polish breed, approximately 8 weeks old and weighing ca. 20 kg. The animals were kept under regular lighting conditions in a temperature—controlled environment. They were fed by commercial grain mixture and tap water *ad libitum*. All the animals were preanesthetized with azaperone (Stresnil, Jansen Pharmaceutica N.V., Belgium, 4 mg/kg of body weight, i.m.) 15 min before the application of the main anaesthetic, sodium thiopental (Thiopental, Sandoz, Kundl-Rakusko, Austria; 10 mg/kg of body weight, given intravenously). In order to localize the sympathetic cell bodies, the pigs were subjected to median laparotomy and received injections of the fluorescent retrograde neuronal tracer Fast Blue (FB, EMS-CHEMIE, GmbH, Germany) into the diamond-shaped part (ca. 4 cm x 4 cm) of the stomach anterior prepyloric wall at a total volume of 50 μl of a 5% solution (1 μl per 1 injection). To minimize leakage of the tracer into surrounding tissues, the needle of the Hamilton syringe was left in place for 20s after each injection, and thereafter the injection area was subsequently rinsed with isotonic saline and gently wiped with gauze. After the surgery injections of the antibiotic (Betamox L.A., ScanVet, Poland, 15 mg/kg b.w., i.m.) and analgesic drugs (meloxicam—Metacam, Boehringer Ingelheim Vetmedica GmbH, Germany, 0.4 mg/kg b.w., i.m.) were applied. In order to minimize post-surgery pain the meloxicam (Metacam, Boehringer Ingelheim Vetmedica GmbH, Germany, 0.4 mg/kg b.w., i.m.) injections were administrated once a day. The health status of the animals was monitored by veterinarian at least 4 time a day.

Afterwards, the pigs were randomly assigned to one of two experimental group: control (C group, n = 5) and ASA group (n = 5). The animals constituting ASA group, from the seventh day after FB injection, were given acetylsalicylic acid orally (aspirin, BAYER; 100 mg/kg b.w.), 1 h before feeding. Gastroscopic examination was performed to exclude lesions in the gastric mucosa in animals from ASA group in the first day and to confirm the gastritis caused by ASA—treatment in the last day of aspirin supplementation (using a video-endoscope Olympus GIF 145 with working length 1030 mm and diameter 9.8 mm).

After 4-week survival time (21^st^ day of ASA treatment), both control and ASA pigs were deeply reanaesthetized and sacrificed by an overdose of sodium thiopental. Then, they were transcardially perfused with 4% buffered paraformaldehyde (pH 7.4). Gastritis in animals of ASA group was confirmed by histopathological examination of fragments of the prepyloric stomach wall collected after perfusion (using routine histopathological methods). Subsequent to perfusion, the following tissues were collected: the coeliac-cranial mesenteric ganglion complex (CCMG) (known also as the coeliac-superior mesenteric ganglion complex (CSMG)); thoracic, lumbar and sacral sympathetic chain ganglia (SChG), cranial and middle cervical ganglia, suprarenal ganglia, small ganglia of the intermesenteric and renal plexuses, caudal mesenteric ganglia (CaMG). Collected tissues were post-fixed by immersion in the same fixative for 20 minutes, rinsed in phosphate buffer (pH 7.4) over three days and finally have been stored in 30% buffered sucrose solution until they sank to the bottom of the container for further processing.

### Immunohistochemistry and statistics

Fourteen-micrometre-thick cryostat sections of the tissue samples were analysed under a fluorescent microscope (Olympus BX 51, Olympus, Poland), equipped with a filter set suitable for observation of FB, to localize and count sympathetic neurons containing the tracer. To determine the relative number of FB-positive perikarya, the neurons were counted in every fourth section. Only neurons with the clearly visible nucleus were considered. Then, selected sections with FB-labelled perikarya were processed for routine double-labelling immunofluorescence technique, using primary antisera raised in different species and species-specific secondary antibodies (Table 1). Briefly, after air-drying at room temperature for 45 min. and rising in 0.1 M phosphate-buffered saline (PBS, pH 7.4; 3 x 10 min.), the sections were blocked with a mixture containing 10% horse serum and 0.1% bovine serum albumin in 0.1 M PBS, 1% Triton X-100, 0.05% Thimerosal and 0.01% sodium azide for 1 h at room temperature to reduce nonspecific background staining. After rinsing in PBS (3 x 10 min.), the sections were incubated overnight at room temperature with primary antisera raised against tyrosine hydroxylase (TH), dopamine β-hydroxylase (DβH), neuropeptide Y (NPY), galanin (GAL), neuronal nitric oxide synthase (nNOS), leu 5-enkephalin (LENK), cocaine- and amphetamine- regulated transcript peptide (CART), calcitonin gene-related peptide (CGRP), substance P (SP) and vasoactive intestinal peptide (VIP) (Table 1). Following subsequent rinsing in PBS (3 x 10 min.), the sections were incubated with mixture of secondary antibodies (Table 1) for 1 h at room temperature to visualize primary antibodies used in this study. After staining, sections were mounted with carbonate-buffered glycerol (pH 8.6) and cover-slipped.

**Table 1:** Specification of immunoreagents.

Antigen	Host species	Code	Dilution	Manufacturer/Supplier
**Primary antibodies**				
**TH**	Mouse	MAB 318	1:200	Millipore, USA
**DβH**	Rabbit	AB1585	1:500	Millipore, USA
**NPY**	Rabbit	NA1115	1:1000	Biomol, Germany
**GAL**	Rabbit	AB2233	1:2000	Millipore, USA
**nNOS**	Rabbit	AB5380	1:2500	Millipore, USA
**LENK**	Rabbit	4140–0355	1:500	AbD Serotec, UK
**CART**	Rabbit	H-003-61	1:15000	Phoenix Pharmaceuticals, USA
**CGRP**	Rabbit	AB5920	1:1500	Millipore, USA
**SP**	Rat	450–0505	1:200	AbD Serotec, UK
**VIP**	Rabbit	PEPA41T	1:1500	AbD Serotec, UK
**Secondary antibodies**				
**Alexa Fluor 488 nm donkey anti- mouse IgG**		A21202	1:1000	Invitrogen, USA
**Alexa Fluor 546 nm goat anti- rabbit IgG**		A11010	1:1000	Invitrogen, USA
**Alexa Fluor 546 nm goat anti- rat IgG**		A11081	1:1000	Invitrogen, USA

Standard controls, i.e. preabsorption for the neuropeptide antisera with appropriate antigen (20 μg of antigen/ml diluted antiserum, all antigens purchased from Peninsula, Sigma or AbD Serotec) and the omission as well as the replacement of all primary antisera by non-immune sera were performed to test the immunohistochemical labelling. There was no fluorescence observed in all these control stainings, what confirms the specificity of the methodology and antibody applied.

Sections were then examined under an Olympus BX51 microscope, equipped with filters suitable for AlexaFluor 488, AlexaFluor 546, and Fast Blue, and pictures were captured by a digital camera connected to a PC, equipped with Olympus Cell F image analyzing software (Olympus, Tokyo, Japan). The sections stained for the same combination of antigens assigned to quantitative investigations were separated by at least 100 μm, to avoid double-analysis of neuronal somata. The number of the FB-positive perikarya counted for every combination of antibodies was above 200 neurons per animal. The data from both groups were pooled, statistically analysed using Statistica 10 software (StatSoft Inc., Tulsa, OK, USA), and were presented as a means ± standard error of mean (SEM). Significant differences were evaluated using the Student’s t test for independent samples (*P <0.05 and **P<0.001). Furthermore, for semi-quantitative evaluation of the density of nerve fibres immunoreactive to each substance studied, an arbitrary scale was used, where (−)—absence of fibres; (+)—single fibres; (++)—rare nervous fibres; (+++)—depicts a very dense nerve fibres.

## Results

### Fast Blue tracing and immunohistochemistry

Fast Blue- containing nerve cell bodies were found exclusively in the CCMG complex, while the rest of pre- and paravertebral sympathetic ganglia were devoid of FB- labelled cells. On average, the CCMG complex contained 1615 ± 20.73 FB+ neurons in the control and 1644 ± 38.03 neurons in the ASA group, respectively. The majority of labelled neurons were located in the region of celiac poles of the CCMG complex, only single- labelled somata in another part of CCMG have been identified. Cell bodies were oval, round or multipolar with short dendrites and centrally situated nucleus and measured 20–45 μm in diameter. In some cases a single long axon-like process traced from the cell were observed.

**Fig 1:**
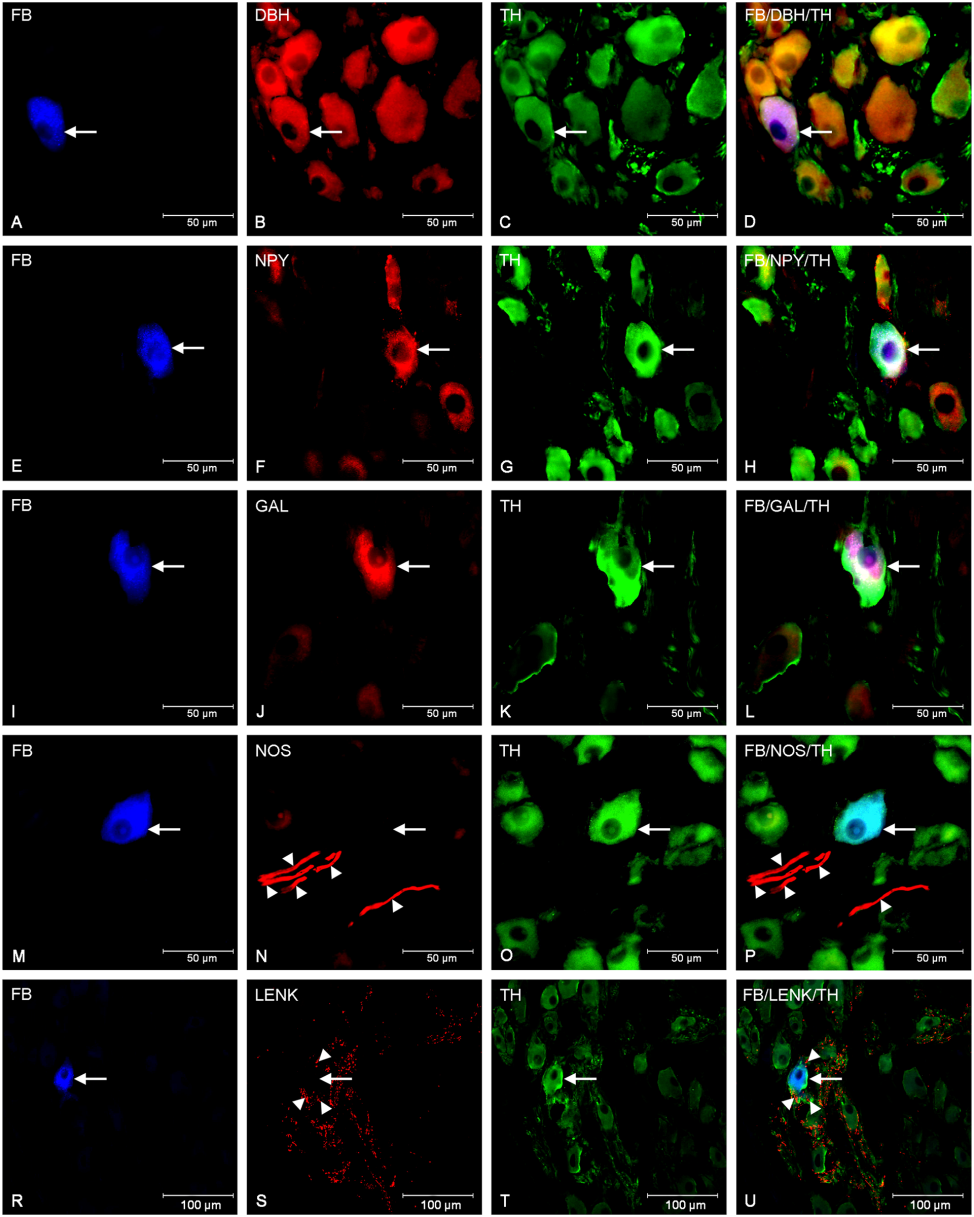
Immunohistochemical characteristic of traced neurons in animals of control group. Fluorescent micrographs showing FB-labelled neurons (A, E, I, M, R) in the porcine CCMG of control animals simultaneously immunoreactive to DβH (B) and TH (C), NPY (F) and TH (G), and GAL (J) and TH (K). Photograph N shows nNOS- IR fibers (arrowheads) in close proximity to the FB- labelled neurons whereas photograph (S) revealed a dense network of LENK-IR fibers (arrowheads) surrounding FB-labelled somata. Photographs D, H, L, P, and U have been created by digital superimposition of three colour channels.

**Fig 2:**
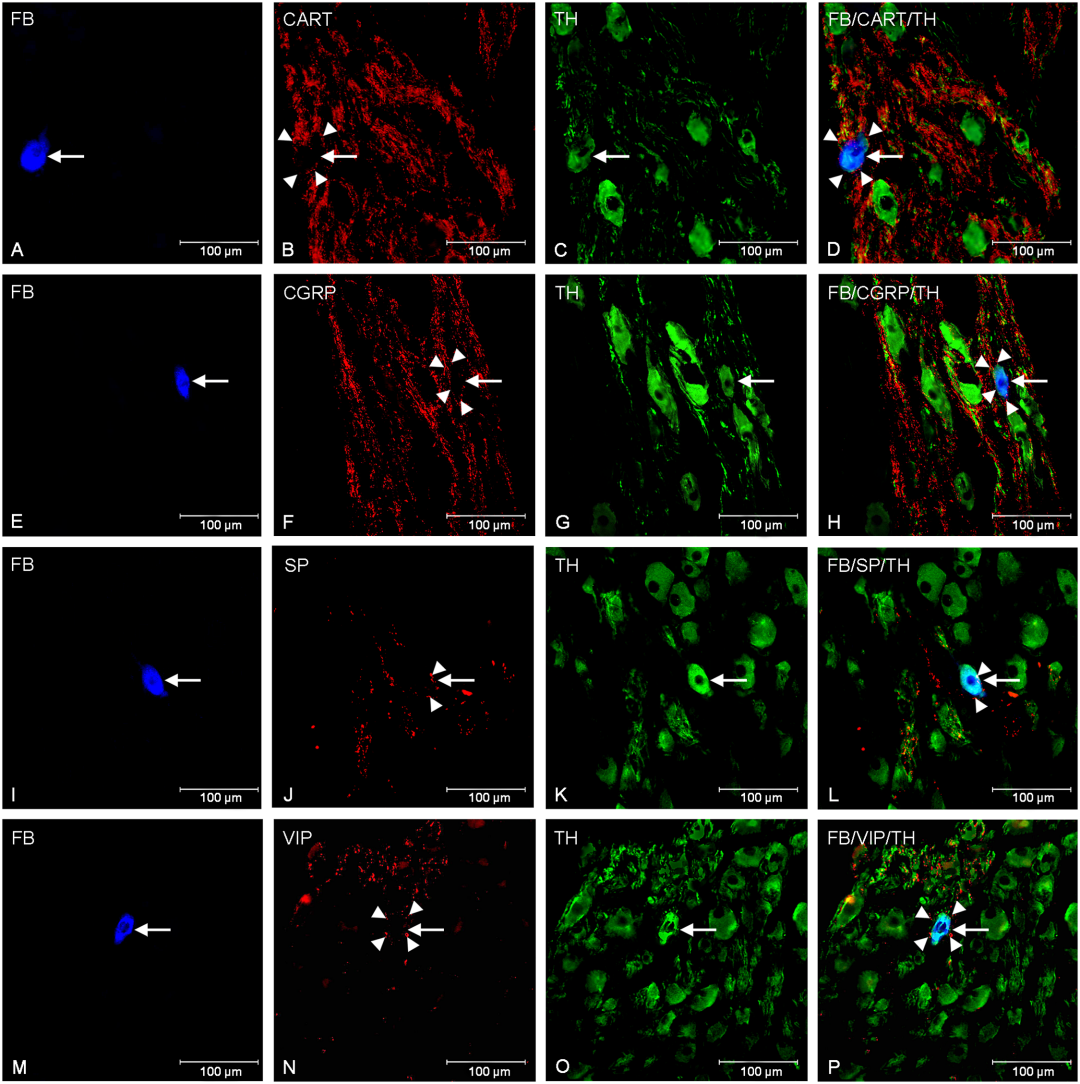
Nerve fibres surrounding traced neurons in animals of control group. Fluorescent micrographs showing TH (C, G, K, O, T) immunoreactivity in the FB-labelled (A, E, I, M) CCMG neurons of control animals and CART- IR (B), CGRP- IR (F), SP- IR (J), VIP-IR (N) nerve fibers (arrowheads) in close proximity of the FB-labelled cell bodies. Photographs D, H, L and P have been created by digital superimposition of three colour channels.

In the control animals double-labelling immunohistochemistry revealed that the great majority of the FB+ cells were immunoreactive to TH (94.85 ± 1.01%) and DβH (97.10 ± 0.97%). Additionally, all TH- positive cell bodies were immunoreactive to DβH what confirmed their strongly catecholaminergic character (Fig 1A, 1B, 1C and 1D). The application of antibodies against neuropeptide Y depicted, that NPY immunolabeling was found in 46.88 ± 2.53% of the FB- labelled perikarya (Fig 1E, 1F, 1G and 1H). In addition, another population of FB- positive neurons contained GAL in 8.40 ± 0.53% (Fig 1I, 1J, 1K and 1L). Moreover, GAL-positive nerve fibers (+) incidentally formed formations encircling GAL- IR cell bodies. On the other hand, none of the FB+ neurons contained nNOS, LENK, CART, SP and VIP. However, all of these neurotransmitters were present in the nerve fibers surrounding the traced perikarya. A large number of varicose CART-positive nerve fibers (+++) closely surrounded the FB-labelled neurons and formed a basket-like structures around them (Fig 2A, 2B, 2C and 2D). Microscopic analysis of sections incubated with anti-CGRP antibodies revealed the dense network of the CGRP-positive fibers (+++) frequently localized nearby the FB-positive cells (Fig 2E, 2F, 2G and 2H). Only single varicose or plain nNOS-IR (+) (Fig 1M, 1N, 1O and 1P), LENK-IR (++) (Fig 1R, 1S, 1T and 1U), SP-IR (+) (Fig 2I, 2J, 2K and 2L) and VIP-IR (+) (Fig 2M, 2N, 2O and 2P) nerve fibers, sometimes forming small bundles, were dispersed throughout the ganglion between traced cell bodies (Table 2).

**Table 2:** Density of nerve fibers surrounding FB-positive perykarya in CCMG complex immunoreactive for specific substances.

Substance	Density of nerve fibres
CART	+++
CGRP	+++
LENK	++
GAL	+
nNOS	+
SP	+
VIP	+
NPY	-
DβH	-
TH	-

**Fig 3:**
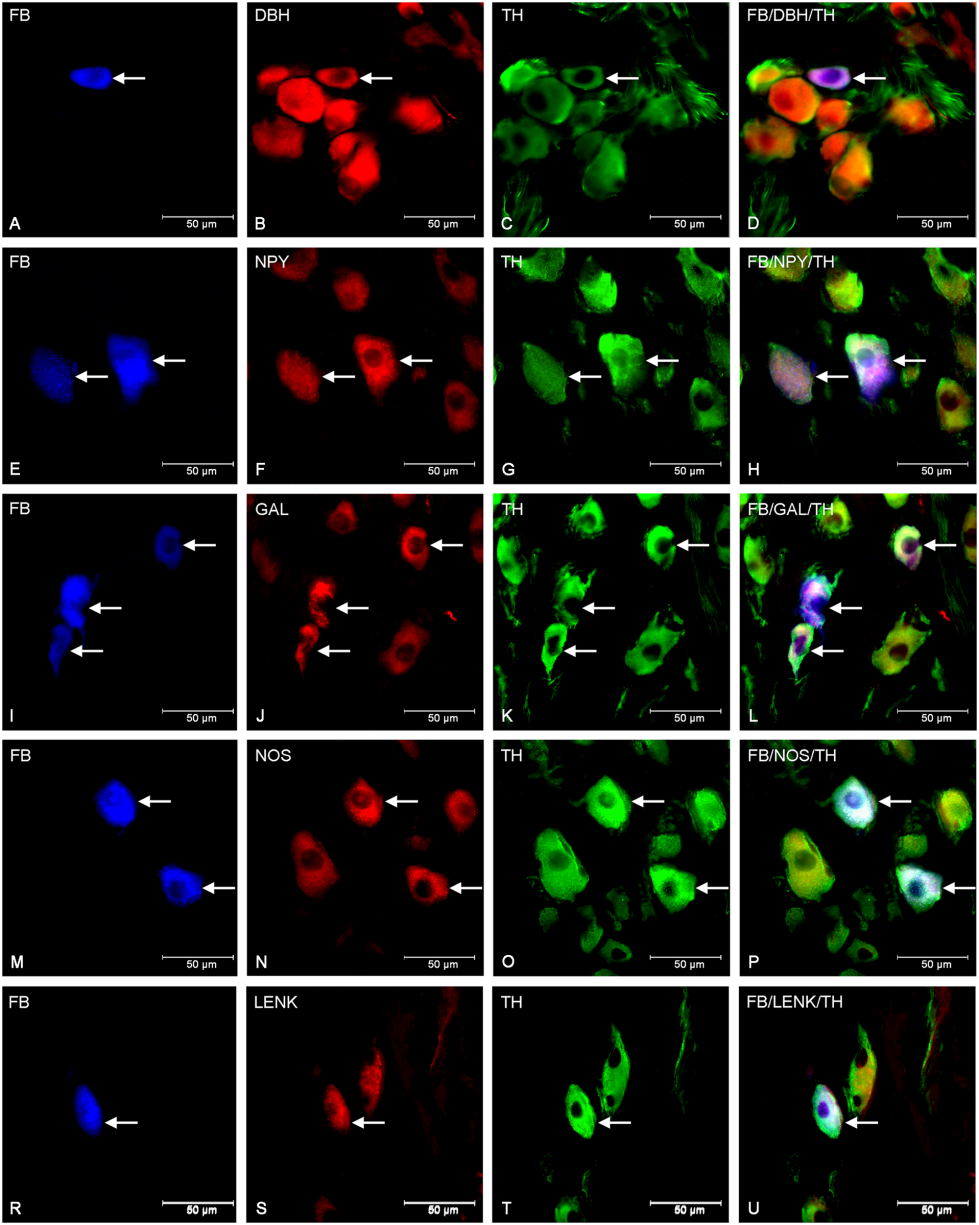
Immunohistochemical characteristic of traced neurons in animals of ASA group. Fluorescent micrographs showing changes in immurectivity of FB-labelled neurons (A, E, I, M, R) in the porcine CCMG of ASA- treated animals. Photographs C, G, K, O and T show neurons immunoreactive to TH and simultaneously to DβH (B), NPY (F), GAL (J), nNOS (N) and LENK (S). Photographs D, H, L, P, and U have been created by digital superimposition of three colour channels. Single perikarya containing TH/DβH (B, C) in contrast to two in control group (Fig 1B and 1C) were visible. Moreover increase in the number of GAL- IR (J) and NPY-IR (F) neurons was observed and the novo-synthesis of nNOS (N) and LENK (S) was detected.

Gastritis induced by prolonged acetylsalicylic acid supplementation changed the coding patterns of many FB+ cells. The population of TH-positive and DβH-positive cells was reduced (Table 3). Microscopic examination of sections showed that 85.78 ± 2.65% were TH-positive, whereas 88.82 ± 1.63% of FB- traced neurons expressed DβH immunoreactivity (Fig 3A, 3B, 3C and 3D). Furthermore, up-regulation of the NPY-IR neurons to 76.59 ± 3.02% was also statistically significant (Fig 3E, 3F, 3G and 3H). The most remarkable difference in the chemical coding of traced sympathetic neurons between the control and ASA—treated pigs included a very increased number of GAL (up to 26.45 ± 2.75%) (Fig 3I, 3J, 3K and 3L). The FB- positive cells containing GAL were also supplied by numerous, mainly varicose GAL-IR nerve fibers. Furthermore, cells containing nNOS in 6.13 ± 1.11% (Fig 3M, 3N, 3O and 3P) and LENK in 4.77 ± 0.42% (Fig 3R, 3S, 3T and 3U) were observed only in ASA- treated animals. Similar to control animals, traced perikarya were not immunoreactive to CART, CGRP, SP and VIP but nerve fibers containing these neurotransmitters were observed in close proximity of the FB- labelled somata and resembled those observed in the control group.

**Table 3:** The average percentage of FB-positive CCMG neurons containing TH, DβH, NPY, GAL, nNOS and LENK in control and ASA group.

	Control	ASA
**TH**	94.85 ± 1.01%	85.78 ± 2.65% *
**DβH**	97.10 ± 0.97%	88.82 ± 1.63% *
**NPY**	46.88 ± 2.53%	76.59 ± 3.02% **
**GAL**	8.40 ± 0.53%	26.45 ± 2.75% **
**nNOS**	0%	6.13 ± 1.11% **
**LENK**	0%	4.77 ± 0.42% **

Data were expressed as mean ± standard error of mean (SEM). The significance of differences was estimated using the Student’s t test for independent samples *P<0.05; **P<0.001.

### Gastroscopic and histopathological examination

**Fig 4:**
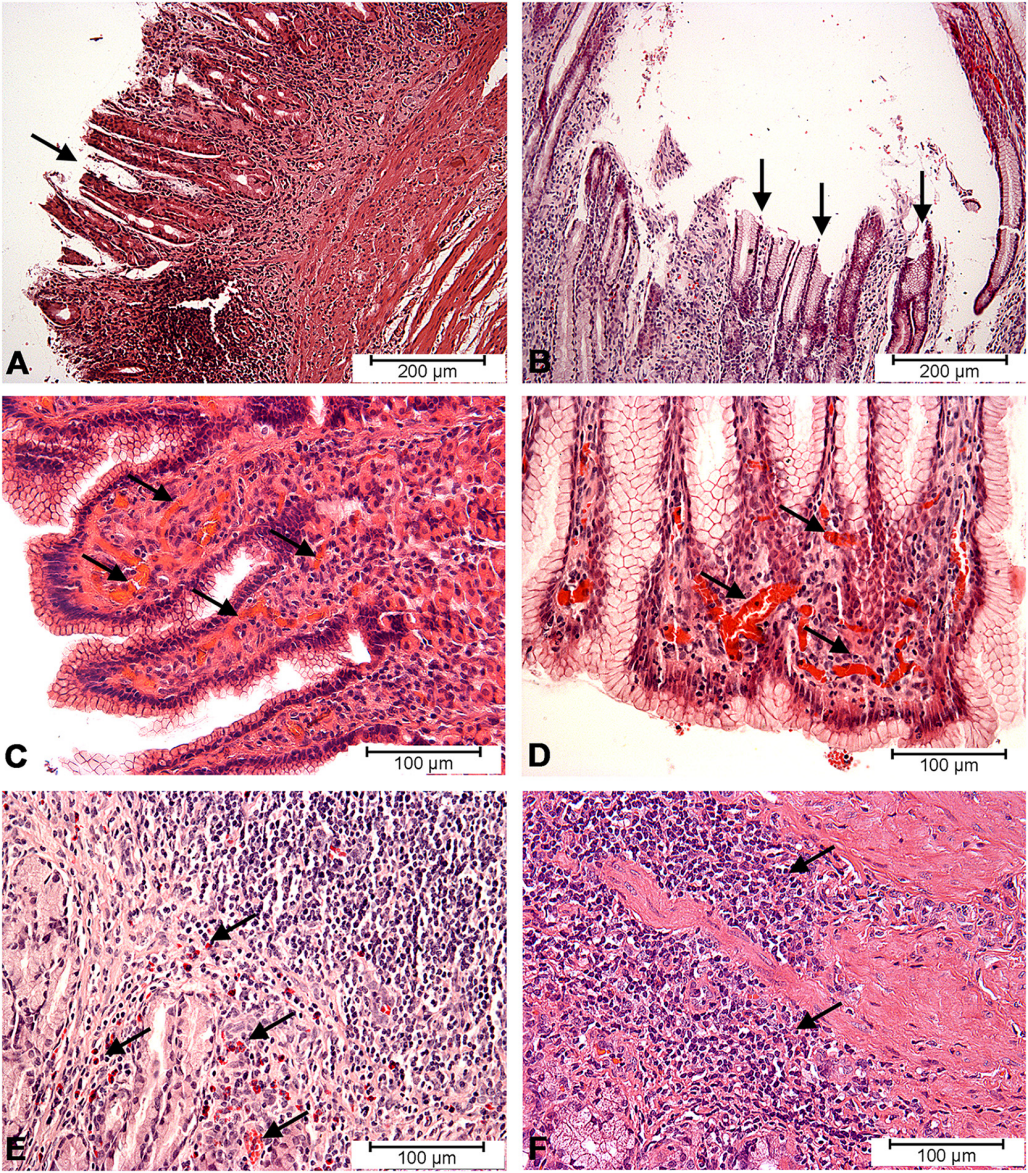
Histopathological changes in gastric mucosa. Histopathological changes in the gastric mucosa membrane caused by acetylsalicylic acid supplementation: superficial erosions (arrows) of gastric mucosa (A, B), hyperaemia (arrows) in gastric mucosa (C, D), infiltration of eosinophils (arrows) in gastric mucosa (E) and prolipheration of lymphatic cells (arrows) in gastric mucosa (F).

Gastroscopic examination performed on the first day of the experiment excluded lesions in the gastric mucosa in animals from both control and ASA group. However, the same examination performed on the last day confirmed the gastritis caused by acetylsalicylic acid supplementation. Macroscopic changes such as: hyperaemia, petechia, superficial erosions and small ulcers were observed not only in gastric, but also duodenum mucosa. Histopathological evaluation of fragments of the wall of gastric prepyloric region disclosed microscopic changes such as: hyperaemia of the mucosa, deep erosions, foliculosis, proliferation of neutrophiles and eosinophilic infiltration extending into the submucosa (Fig 4A, 4B, 4C and 4D).

## Discussion

This is the first report demonstrating exact localization, morphology and chemical coding of sympathetic neurons projecting to prepyloric area of the porcine stomach. The major source of postganglionic nerve fibers supplying this region originate from the CCMG complex, while the pre- and paravertebral sympathetic ganglia were devoid of the FB- labelled cells. These results are consistent with the data obtained from rat [3, 12], guinea pig [15, 16], and dog [7] and provide further evidence establishing the predominant importance of CCMG neurons in stomach innervation. Notwithstanding, minor input in sympathetic innervation of stomach in rat and guinea pig came from thoracic chain ganglia. Neurons supplying gastric wall in the rat were located from T_1_ to L_3_ in the right chain, and from T_2_ to L_3_ in the left respectively [10], whereas cell bodies innervating rats gastric mucosa were found from T_6_ to T_9_ sympathetic chain ganglia [3]. In the guinea pig neurons located in all thoracic chain ganglia were projecting to stomach [16]. In contrast, this research shows that these ganglia are not involved in the innervation of prepyloric region of the porcine stomach, most likely they innervate other parts of stomach, which requires further investigation. Moreover, this findings correlate well with those studies postulating characteristic topographical arrangement of neurons, with respect to their target tissues, in the porcine CCMG complex [43].

The present study has disclosed that the great majority of FB—positive neurons in control group had strongly catecholaminergic character, as revealed by simultaneous TH- and DβH- immunoreactivity. These results correlate well with studies describing adrenergic nature of most CCMG neurons in the rat, guinea pig, dog and domestic pig [10, 16, 44, 45]. Only small number of traced CCMG neurons was devoid of these enzymes, which may indicate, that they are population of non-adrenergic, possibly cholinergic neurons, which is in agreement with previous findings on histochemistry of CCMG in both guinea and domestic pigs [45, 46]. Moreover, this data indicate that significant amount of FB- labelled perikarya expressed NPY immunoreactivity. Literature data suggest that NPY is considered to be the major peptidergic transmitter in both: the sympathetic and parasympathetic nervous systems [47]. Namely, NPY as neuronal and hormonal regulator play important role in mammalian physiology of gastrointestinal tract, including inhibition of gut motility, gastric emptying, acid secretion and pancreatic exocrine secretion. Moreover, this peptide affects blood vessels and participates in regulation of intestinal blood flow [48, 49]. On the other hand, NPY was also observed in porcine CCMG neurons projecting to ileum [50]. Further, this study revealed that GAL- IR neurons represent portion of the retrogradely labelled cells. It well correlates with fact that galanin is biologically active neuropeptide, having a widespread distribution in the central and peripheral nervous systems as well as peripheral tissues of many species [51–53]. At the gastrointestinal level, galanin inhibits gastric acid secretion, the release of numerous pancreatic peptides, regulates mucosal epithelial cell absorption and modulates motility of gastrointestinal tract [54]. Galanin exerts its physiological role by acting on one of the three different subtypes of G protein-coupled receptors (GAL-R1, GAL-R2 and GAL-R3) widespread in many tissues and organs [52].

Although none of FB- positive neurons contained nNOS, LENK, CART, CGRP, SP and VIP, all of these neurotransmitters were present in the nerve fibers surrounding the traced perikarya. The numerous varicose CART-positive nerve fibers closely surrounded the FB-positive neurons and formed a basket- like structures around them. Cocaine- and amphetamine-regulated transcript (CART) peptide discovered in 1981 in ovine hypothalamus has been described in various segments of the GI tract of numerous mammalian species, including humans [2, 55, 56]. Despite the fact that the role of this peptide in gut function is not fully established, some authors suggest that CART is involved in the regulation of intestinal motility, inhibition of feeding, reduction of gastric acid secretion, exacerbation of colonic motility [57]. Hitherto, changes in the number of CART-positive nervous structures in the ENS under various pathological factors were described, what might suggest involvement of CART in survival and neuroprotection [58, 59]. CGRP and SP are considered as pronociceptive sensory neuropeptides [60]. The number and distribution of SP-IR and CGRP-IR fibers are quite similar to those that have been already described by Lakomy et al. [45] for the fibers surrounding TH-positive neurons in the porcine CCMG. They represent one of the sources of the viscerofugal projections from the gut wall or originate from DRG afferent neurons, because these specifically coded neurons were described in enteric ganglia [55, 61] as well as are widely distributed in sensory neurons [5]. Moreover, single varicose VIP-IR nerve fibres dispersed throughout the ganglion between traced cell bodies were observed. VIP is probably involved in an intestine—intestinal inhibitory reflex arc and occurs in enteric afferents innervating the CCMG [45]. Finally, source of the nNOS-IR and LENK-IR fibers visualized in this study could be the preganglionic neurons located in the intermediolateral nucleus of the lumbar spinal cord or from DRG neurons. The close proximity of fibers disclosed in this study in relation to retrogradely labelled CCMG neurons may indicate the indirect effects of this peptide in the sympathetic innervation of the stomach. However, the question of the impact of these fibers on sympathetic neurons supplying prepyloric area of stomach remains to be elucidated.

Despite the fact that inflammation of the gastric mucosa, caused by long-term acetylsalicylic acid supplementation, has no influence on the number of neurons innervating the studied area of stomach, retrogradely labelled sympathetic cells exhibit a great deal of plasticity in their neuropeptides phenotype. ASA-induced gastritis led to the significant changes in the chemical coding of traced neurons, by reducing production of the catecholamine-synthesis tract enzymes and up-regulation of synthesis of neuropeptides involved in neuronal defence mechanisms (NPY, GAL, nNOS, LENK). These data are in agreement with the fact that regenerating sympathetic neurons temporarily down-regulate the expression of some neurotransmitters especially TH [32] and start to produce neurotransmitters involved in the defence and survival [31].

NPY is emerging as regulator of inflammation, involved in autoimmunity, asthma, cancer and many gastrointestinal disorders, such as melabsorption, short gut, inflammatory bowel disease and pancreatitis. NPY, as an anti-inflammatory peptide, participates in the inflammatory response by the recruitment of immature dendritic cells and by promoting a Th2 polarization [49]. NPY can also be involved in inflammatory response through differentiation of T helper cells, monocyte mediator release, natural killer [NK] cell activation, and immune cell redistribution what confirmed earlier reports of bidirectional crosstalk between nervous and immune systems in gastrointestinal tract [62, 63]. These findings are similar to those of previous studies, where the number of NPY-positive sympathetic neurons were elevated during various pathological conditions, including proliferative enteropathy and porcine colitis [4, 28]. Furthermore, experiment in human depicted an increase in NPY- IR nerve endings within the mucosa during chronic gastritis caused by *Helicobacter pylori* [62]. This suggest that NPY is important neuroprotective factor, which plays the role in survival and regeneration of damaged neurons within inflammation. Interestingly, NPY can also be secreted by some immunocytes, which may suggest that NPY is directly involved in neurogenic inflammation [64]. However, the exact function of NPY in neuronal response during ASA-induced gastritis in pigs remains unexplained and requires further investigation.

The most remarkable difference in immuhistochemical phenotype of traced neurons was a very increased number of GAL- immunoreactive cells bodies in ASA- group animals. Galanin is unquestionably engaged in the regulation of inflammatory processes. Indeed, galanin is considered as a regulator of pro-inflammatory cytokines, because the administration of galanin has increased production of TNF-α, IL-1α and IL-8 in human keratinocytes in physiological state [65]. To date, the increases in galanin expression in neural structures, both autonomic and enteric nervous systems, were observed during chemically induced colitis [4, 51] and proliferative enteropathy [28] in pigs, chronic diverticulitis in human [66] and enteric Salmonella infection in mice [67]. Interestingly, Talero et al. [68] reported that chronic administration of galanin exhibits an anti-inflammatory activity in the experimental trinitrobenzensulfonic acid (TNBS) acute model of inflammatory colitis by reducing the NOS expression and activity as well as with a possible participation of mucosal mast cells. Additionally, elevated expression of galanin receptors, especially GalR1, was noted in peripheral tissues of every experimental inflammatory model [54]. Notwithstanding, that galanin seems to play protective function for damaged neurons, in cell culture it decreased neuronal survival [69].

nNOS is an indicator of nitric oxide (NO) prevalent in the neurons of the central and autonomic nervous systems [70]. NO produced by calcium-dependent NOS play a major role in gut mucosal protection and motility [71]. Elevation of the number of nNOS–expressing neurons under various inflammatory processes and axotomy has also been observed by many authors [29, 72]. These data revealed that CCMG neurons showing expression of nNOS were found only in animals of the ASA group. It well corellates with the fact that NO is considered as an inflammatory factor involved in acute and chronic inflammatory responses [73]. NO has many beneficial effects during inflammation such as: protection of mucosa, prevention of cellular damage, regulation of mucus secretion, mucosal blood flow, reparation of ulcers or influence on mucosal immunocytes activity [74, 75]. Moreover, NO presents gastroprotective properties against different types of aggressive agents and the inflammation is reduced by inhibiting the activation of nuclear factor-kappaB [76]. The results of present study and these facts are congruent with recent study on protective influence of NO in nonsteroidal anti-inflammatory drugs (NSAID)—gastroenteropathy, and confirm that NO donors may be a potentially useful drugs acting as a retardants for gastrointestinal mucosa in the long-term NSAID therapy [77].

Leu 5-enkephalin (LENK) is an endogenous opioid peptide neurotransmitter detected within gastrointestinal tract in endocrine cells and neurons of ENS, as well as extrinsic neurons [55]. Experiments in rats depicted that endogenous opioids are released within certain brain area in response to different stressful stimuli and modulate nociception. Furthermore, LENK may be secreted by leukocytes and by activation of sensory opioid receptors and may lead to inhibition of local inflammatory pain [78]. Results of de novo-synthesis of LENK in CCMG neurons in ASA- induced gastritis may implicate this opioid in neuronal response to inflammatory process. Indeed, sympathetic and sensory nerve fibers up-regulate the expression of vascular endothelial ICAM-1 leading to activation of opioid peptide-containing leukocytes and reduce inflammatory pain [78]. However, some of the experimental studies in various animal models of inflammation exhibit the decreased expression of LENK in neuronal and non-neuronal structures [28, 79]. Although the literature data and results of this study suggest that LENK is involved in the regulation of inflammatory pain, supplementary ultrastructural and functional investigations are required in order to explain these mechanisms.

In conclusion, the obtained data showed that postganglionic sympathetic nerve fibers supplying prepyloric area of the porcine stomach originate from the CCMG complex. Retrogradely traced neurons contained TH, DβH, NPY and GAL, what may suggest their involvement in sympathetic regulation of the stomach function. ASA- induced gastritis resulted in increased expression of NPY and GAL, as well as the novo-synthesis of nNOS and LENK in traced CCMG neurons. The results of present study indicate involvement of this neuropeptides in the development and presumably counteraction of gastric inflammation. Additionally, the network of CART-, CGRP-, SP-, VIP-, LENK-, nNOS- IR nerve fibers encircling/neighbouring FB- positive perikarya, observed in both intact and ASA- treated animals, suggests that these neuropeptides may have the role as indirectly acting transmitters as well as modulators of sympathetic control of gastric function in the pig.
